# Seroprevalence and Geographical Distribution of Rift Valley Fever in Small Ruminants in Mauritania: Evidence of Endemic Circulation and Regional Risk Hotspots

**DOI:** 10.3390/v18070722

**Published:** 2026-06-30

**Authors:** Abdellahi El Ghassem, Mariem Seyidna Khayar, Mariem Cheikh Ahmed, Ekatrina Isselmou, Abdellahi Diambar Beyit, Barry Yahya, Yacoub Sidi Moctar, Mohamed Baba Gueya, Navaa Abdelawahab, Habiboullah Habiboullah, Sébastien Briolant, Ahmed Bezeid Ould El Mamy, Ali Ould Mohamed Salem Boukhary

**Affiliations:** 1 Unite de Recherche Génomes et Milieux (GEMI), Faculté des Sciences et Techniques, Université de Nouakchott, Nouakchott B.P. 5026, Mauritania; abdellahielghassem@gmail.com; 2 Office National de Recherches et du Développement de l’Élevage et du Pastoralisme, Nouakchott B.P. 167, Mauritania; ksmaou11@yahoo.fr (M.S.K.); mariembegnoug@gmail.com (M.C.A.); katiaisselmou@gmail.com (E.I.); jembar2000@yahoo.fr (A.D.B.); barryyahya07@gmail.com (B.Y.); y.sidimoctar@gmail.com (Y.S.M.); ouldnet@yahoo.fr (M.B.G.); 3 Direction des Services Vétérinaires, Nouakchott B.P. 115, Mauritania; wehab.nava@gmail.com (N.A.); habiboullahdsv@gmail.com (H.H.); 4 Unité Parasitologie et Entomologie, Département Risques Vectoriels, Institut de Recherche Biomédicale des Armées (IRBA), 13005 Marseille, France; 5 Unité Mixte de Recherche Risques Infectieux Tropicaux et Microorganismes Emergents, Assistance Publique des Hôpitaux de Marseille, Service de Santé des Armées, Aix Marseille University, 13005 Marseille, France; 6 Institut Hospitalo-Universitaire, Méditerranée Infection, 13005 Marseille, France; 7 Institut Supérieur d’Enseignement Technologique (ISET), Rosso B.P. 172, Mauritania; bezeid07@yahoo.fr

**Keywords:** Rift Valley fever, sheep, goats, seroprevalence, ELISA, Mauritania

## Abstract

Rift Valley fever (RVF) is a mosquito-borne viral zoonosis that causes severe illness in livestock and humans, with significant economic and health repercussions. Mauritania is considered an RVF focus in West Africa. A cross-sectional survey was conducted in 2023 in 12 of Mauritania’s 15 provinces. Serum samples from 849 small ruminants (428 goats and 421 sheep) were analyzed by ELISA for IgG and IgM antibodies against RVF virus. Logistic regression analyses were performed to assess associations between seropositivity and species, age, sex, season, and geographic location. In total, 14.1% of the animals tested were positive for anti-RVFV IgG antibodies. There was no association between anti-RVFV IgG positivity and the ruminant species, the sex, the age and the season. Hodh El Gharbi had the highest seroprevalence (40.5%), followed by Adrar (19.8%) and Tagant (19.7%). The lowest levels were recorded in Tiris Zemmour (2%) and Inchiri (3%). Only two animals tested positive for IgM, suggesting limited recent viral activity. This nationwide survey confirms widespread exposure of small ruminants to RVFV in Mauritania. Strengthening longitudinal serological monitoring and integrating ecological and entomological data within the “One Health” approach will be essential to preventing future epidemics and protecting animal and human health.

## 1. Introduction

Rift Valley Fever (RVF) is caused by a virus belonging to the genus *Phlebovirus* which is mainly transmitted to domestic and wild animals through the bites of several species of mosquitoes, including those of the genera *Aedes* and *Culex*. The virus can also be transmitted to humans through direct contact with the blood, body fluids, or tissues of infected animals [1]. Rift Valley fever virus (RVFV) can cause severe illness in ruminants and humans, with significant repercussions for public health and economic systems [2]. In ruminants, RVFV infection is characterized by waves of abortions and neonatal mortality rates of up to 100% [3].

In Mauritania, cases of RVF have been documented since the 1980s [4], although modeling studies suggest earlier viral circulation [5]. The virus re-emerges periodically, approximately every 4.5 years, causing epizootics and epidemics, particularly during or at the end of the rainy season [6]. The number of reported human cases of RVF varies considerably from year to year [6]. However, the country is one of the main foci of RVF in West Africa [4] and the disease represents the most common hemorrhagic fever at the national level [6]. Ecological factors are often associated with the occurrence of RVF in Mauritania, such as the construction of dams or the presence of exceptionally rainy years [7]. The strategy for monitoring RVF in Mauritania relies primarily on active entomological surveillance conducted during the rainy season and the deployment of sentinel herds across the country. These herds are monitored at regular intervals during the period from August to October, corresponding to the rainy season [8]. The system also includes passive surveillance via a notification system for health centers and the use of sentinel herds for surveillance purposes [9].

Small ruminants, sheep and goats are commonly used as sentinel animals for RVF surveillance in many endemic countries, including Mauritania, because they are highly susceptible to infection, especially young animals. Infected animals often develop severe clinical signs such as waves of abortion and high neonatal mortality, which constitute an early epidemiological signal of virus circulation [1,3,10]. Their short incubation period and rapid immune response make serological surveillance effective, while their abundance, accessibility, and low maintenance costs allow for practical, large-scale surveillance in endemic areas [3,10].

In Mauritania, livestock farming remains the dominant economic activity in most regions, particularly in rural and pastoral zones. It is estimated that 60 to 70% of the population, or approximately 2.5 to 3.5 million people, depend directly or indirectly on livestock farming for their livelihoods [11]. In addition, the country has one of the largest livestock herds in Africa, with 14.6 million sheep, 9.4 million goats, 2.3 million cattle, and 1.5 million camels spread across the entire territory [12]. Consequently, close contact between livestock farmers and their animals is frequent in Mauritania, posing a significant health risk to humans.

Although Rift Valley fever (RVF) is endemic in Mauritania, the actual national baseline seroprevalence and the spatial distribution of the virus across the country’s various ecological zones have never been systematically determined. Previous studies have relied on sporadic, reactive data from active outbreaks, leaving significant gaps in our understanding of the virus’s distribution across different ecological zones during inter-epidemic periods. The objective of this study was to assess the presence of RVFV in small ruminants in Mauritania in order to better understand its persistence in areas likely to become hotspots for disease emergence under favorable conditions.

## 2. Materials and Methods

### 2.1. Ethics Statement

The collection of animal blood samples was carried out by the National Office for Research and Development of Livestock and Pastoralism (ONARDEP) as part of its governmental mandate to monitor and track livestock for veterinary and zoonotic pathogens, in compliance with all applicable national and international regulations and fundamental ethical principles. The experimental protocol for this study was reviewed and formally approved by the Research Ethics Committee of the University of Nouakchott (No. 003-2022-CER). Informed consent for the collection of animal blood samples was obtained from the animal owners.

### 2.2. Study Sites

Mauritania is a vast, almost desert country in West Africa (1,030,700 km^2^), located between 15° and 27° north latitude and 5° and 17° west longitude. Administratively, the country is divided into 15 Wilayas (provinces) comprising 63 Moughataas (departments) and 220 communes [11]. According to the aridity index (AI), Mauritania has three distinct ecological zones: the Saharan zone, which covers the northern two-thirds of the country (hyper arid zone, AI < 0.05), and the Sahelian zone, which occupies the remaining southern third (arid zone, 0.05 ≤ AI < 0.2). A small area in the extreme south of the country belongs to the Sudanian zone (semi-arid zone, 0.2 ≤ AI < 0.5) [13].

In rural areas of Mauritania, agropastoralism plays a central role, providing food for local populations and producing goods for the market. In the northern desert region, the population lives off oases and raises camels and goats. In both the central and southern regions, populations depend on irrigated rainfed agriculture and ruminant livestock farming. The generally hot and dry climate necessitates the construction of dams to retain rainwater and supply the population and agricultural activities. This promotes the establishment of a potential vector of RVF in Mauritania, mainly *Culex* and *Aedes* species [14].

**Figure 1:**
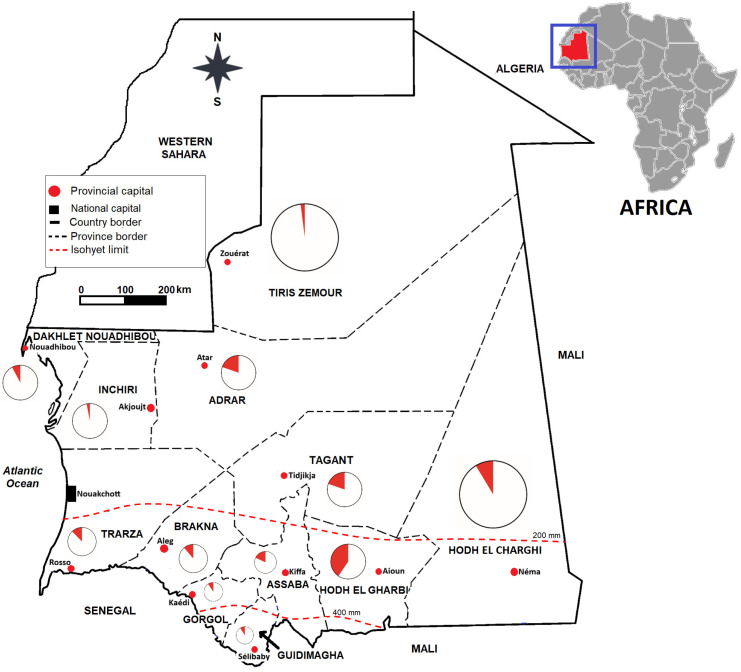
Map of Mauritania showing the study sites and the seroprevalence of Rift Valley fever. Only Nouakchott, the capital administratively divided into three regions, was not included in the present study. Study sites in each administrative region were as follows: Adrar (Atar, Aoujeft); Assaba (Kankoussa, Kiffa, Guérou); Brakna (Aleg, Boghé, Magtaa Lahjar); Gorgol (Kaédi, Maghama, Mbout); Guidimagha (Ould Yenje, Ghabou, Sélibaby); Hodh El Charghi (Adel Bagrou, Amourj, Djiguenni, Néma, Timbedra); Hodh El Gharbi (Tamchekett, Tintane, Kobenni); Inchiri (Akjoujt, Benchab); Dakhlet Nouadhibou (Chami, Nouadhibou); Tagant (Moudjéria, Tidjikja, Tichit); Tiris Zemmour (Bir Moghrein, Zouérat); Trarza (Keur macène, Méderdra, Rkiz). The pie charts show the proportions of animals that are seropositive (in red) and seronegative (in white) for total anti-Rift Valley fever virus IgG.

The study was conducted in 12 of Mauritania’s 15 administrative regions (Figure 1). Nouakchott, with its three administrative regions, was not included in this study. Sampling sites were selected based on the relative proportions of small ruminant herds and the community’s willingness to participate in the study.

### 2.3. Study Design and Animal Sample Size

This was a cross-sectional survey conducted during the dry (January–March) and wet (August–September) seasons of 2023. Stratified sampling was used to select herds, while random sampling was applied to small ruminants within the herds.

For each ruminant species, the minimum number of animals to be sampled for the entire study area was calculated using the following formula:

N = 1.96^2^ × P (1 − P)/D^2^

where N = estimated minimum sample size; P = estimated prevalence (since the national prevalence of RVF had not been determined in the country, we used a priori estimate of 50% to obtain maximum variance); D = 5% precision (with a 95% confidence interval). This process resulted in a minimum sample size of 385 goats and 385 sheep, for a total of 770 small ruminants.

### 2.4. Sample Collection and Processing

A total of 849 small ruminant samples, including 428 goats and 421 sheep, were included in this study. On average, 10 to 15 animals per site were randomly selected from resident small ruminants over seven months of age, with no balance between species. The animals’ ages were provided by the farmers. Suckling or recently weaned kids and lambs (generally under 6 months of age) were excluded from the study due to the possible presence of maternal antibodies. The sampled animals were arbitrarily classified into two groups: those aged 7 to 12 months and those over 12 months of age.

### 2.5. Blood Sample Collection

Blood samples were taken from the jugular vein into dry tubes and left upright on a flat surface until the clot retracted, then the plasma was transferred into cryotubes, kept in a cooler and transported to the National Office for Research and Development of Livestock and Pastoralism (ONARDEP) where they were stored at −20 °C until the serological analysis was conducted.

### 2.6. Serum Sample Laboratory Analysis

#### 2.6.1. Rift Valley Fever Virus IgM ELISA

All serum samples were analyzed by ELISA for recent RVF virus exposure using the ID Screen^®^ Rift Valley Fever IgM Capture kit (ID-Vet Innovative, Grabels, France), according to the manufacturer’s instructions. The test was considered valid if the mean positive control optical density > 0.350 and the ratio of the mean value of the positive and negative control ODs was >3. The sample-to-positive percentage was calculated as S/P% = [net OD of the sample/net OD of the positive control] × 100. Results were interpreted as negative (S/P% ≤ 40%), doubtful (40% < S/P% < 50%), or positive (S/P% ≥ 50%). The sensitivity and specificity reported for this kit range from 94.6% to 100%, and from 95.6% to 99.8%, respectively [15].

#### 2.6.2. Rift Valley Fever IgG ELISA

The ID Screen^®^ RVF multispecies indirect assay kit (ID-Vet Innovative, Grabels, France) was used to detect the presence of IgG, in accordance with the manufacturer’s instructions. The test was considered validated if the mean OD of the negative control was >0.7 and if the mean OD of the positive control met the criterion [OD of the positive control/OD of the negative control < 0.3]. The percentage of competition was calculated using the following formula: S/N % = (OD of the sample/OD of the negative control) × 100. The results were classified as positive (S/N % < 40%), equivocal (40% < S/N % < 50%), or negative (S/N % > 50%). The diagnostic sensitivity and specificity reported for the kit were 98% and 100%, respectively [16].

### 2.7. Statistical Analysis

All collected data was entered, processed and analyzed using Microsoft Office Excel 2016. Descriptive statistics were used to summarize categorical variables such as species, sex, age group, and geographic location. The seropositivity of anti-RVF IgG was modeled by a logistic regression with the geographical location as a random effect using R software (version 4.4.2) [17] and variables with a *p*-value < 0.25 were retained for multivariate analysis as previously reported [18]. A stepwise procedure based on Akaike’s information criterion (AIC) was used to select the final significant variables (*p*-value < 0.05).

## 3. Results

### 3.1. Characteristics of the Studied Population

Table 1 details the geographical origin of the species included in the study and the number of herds sampled per region. In total, 849 serum samples (421 sheep and 428 goats) were collected from 59 sites in Mauritania, excluding the capital, Nouakchott. Overall, the sampled small ruminant populations show a statistically equal distribution (*p* = 0.810) across species. However, goats predominate in Assaba (55 vs. 11), Trarza (70 vs. 11), Inchiri (62 vs. 4) and Tiris Zemour (48 vs. 2) while the ovine species predominates in the sample of Hodh El Gharbi (57 vs. 22), Hodh El Charghi (60 vs. 21), Guidimagha (70 vs. 9) and Dakhlet Nouadhibou (42 vs. 9).

### 3.2. Serology of Rift Valley Fever in Small Ruminants

The study analyzed 849 serum samples for IgG antibodies against RVFV (Table 2). The overall seroprevalence in the ruminants studied was 14.1% (120/849). There was no statistically significant difference (*p* = 0.51) between seroprevalences against RVFV in goats (13.6%) and sheep (14.7%).

Females showed a slightly higher prevalence (14.9%) than males (8.7%), but without statistical significance (*p* = 0.31). Seroprevalence in animals aged 7 to 12 months (11.0%) was not statistically significant (*p* = 0.93) compared to that in animals aged over 12 months (14.5%). The seroprevalence of anti-RVFV IgG showed a very heterogeneous distribution between regions. Hodh El Gharbi had the highest seroprevalence (40.5%, 32/79) followed by Adrar (19.8%) and Tagant (19.7%). Tiris Zemmour (2%) and Inchiri (3%) had the lowest seroprevalences. There was no statistically significant difference (*p* = 0.12) between seroprevalence in the rainy season (16.5%) and in the dry season (11.9%).

It should be noted that only 2 sera were found to be positive for IgM based on ELISA (1 in Hodh El Gharbi and 1 in Tagant).

### 3.3. Serological Status of Aborted Animals

Of the 849 animals included in the study, 15 abortions were recorded. Table 3 shows the regional distribution of these abortions. The proportion of animals that aborted and had a positive competitive ELISA test was 33.3%, while the proportion of animals that tested negative was 66.7%. The highest number of abortions was observed in the Hodh El Gharbi sample, where nearly half the animals were seropositive (4 out of 7). Next was Adrar, which recorded 4 seronegative animals that aborted. In Tagant, two animals aborted, one of which was seropositive. Finally, Hodh El Charghi and Assaba each recorded one abortion in a seronegative animal. All of these abortions occurred in 2023, except for the one in Assaba, which occurred at the end of 2022.

## 4. Discussion

Small ruminants constitute a major economic resource for vulnerable rural households—and increasingly for urban communities—in Mauritania [11]. Given that sheep and goats are widespread, frequently traded and raised in close contact with humans, they constitute a key population for RVF surveillance. Their mobility through transhumance and livestock markets further increases the potential for zoonotic transmission, reinforcing their epidemiological importance [19].

This national seroprevalence survey revealed widespread antibody presence against RVFV in small ruminants in Mauritania. Of 849 samples tested, 120 were positive, representing an overall seroprevalence of 14.1%. This result is higher than that of previous studies, many of which were conducted in epidemic-prone areas or during epidemic periods [20,21]. Our nationwide sampling, conducted during a period of low or undetectable RVF activity, provides a broader and potentially more representative estimate of background exposure.

Only two IgM-positive samples were detected, both during the dry season, while none were found in the sample collected during the rainy season. These IgM-positive cases likely reflect late infections occurring during the previous transmission period, and subsequent follow-up visits confirmed the presence of additional IgM. The overall rarity of IgM-positive cases, combined with the absence of reported outbreaks in livestock, suggests limited recent transmission and persistent IgG antibodies following prior exposure.

Previous studies on the seroprevalence of RVF in Mauritania have shown marked temporal variability [5,22,23]. The first documented survey of RVF (1982–1984), conducted after the construction of dams along the Senegal River, reported a seroprevalence of 15.6% among small ruminants in the southeastern regions [4]. Subsequent epidemics have highlighted a sharp increase in exposure. In 1987, in the Senegal River basin, seroprevalence reached 37% [24,25]. Between 1988 and 1992, it decreased to 18.7% in the same area [26]. In 2003, the central–southern and western regions experienced infection rates of 46% in small ruminants near confirmed human cases [27]. An unexpected outbreak in northern Mauritania in 2010 showed a seroprevalence of 44% [20], rising to 69% by early 2011 [7]. During the inter-epidemic period of 2012–2013, seroprevalence fell to 3.8% [22]. In 2020, a large-scale epidemic was associated with a seroprevalence of 27.9% among suspected cases and 47% among small ruminants living in close proximity to human cases [28]. Our observed seroprevalence of 14.1% is higher than the 3.8% reported during inter-epidemic periods [22] but lower than the levels documented during major epidemics, supporting the notion of cyclical RVF activity influenced by environmental conditions such as extreme flooding (37%) [24] or unusual rainfall (69%) [7].

Although vaccination can induce false-positive diagnostic results particularly in younger animals depending on the vaccine type this confounding factor is negligible in Mauritania. Despite the endemicity of Rift Valley fever (RVF) in the country, a recent joint FAO/WHO/WOAH report on risk assessment of RVF in Senegal and Mauritania highlights an absence of preventive vaccination against RVF in susceptible livestock and notes that immunization is not practiced on a routine basis. Because routine vaccine coverage is virtually nonexistent and herd immunity remains critically low, the risk of vaccine-induced antibody interference is minimal, confirming that our findings reflect genuine natural exposure and active viral circulation [29].

In Mauritania, the emergence of RVF is strongly linked to hydro-ecological and climatic conditions. The disease is generally associated with areas near permanent bodies of water and tends to spread geographically during years of heavy rainfall [7,24]. Conversely, a decrease in rainfall is associated with a lower seroprevalence [22].

Environmental and anthropogenic factors including hydro-ecological changes, seasonal rainfall, livestock movement and human population density, significantly influence the dynamics of RVF [19]. The main emergence period is generally between September and October, which corresponds to the end of the rainy season. Weather anomalies may also explain the northward expansion of RVF, as historically transmission was concentrated in the southern and central regions, but more recent outbreaks have occurred in the northern regions [6].

Antibodies were detected in all studied regions, confirming widespread circulation. The highest seroprevalence was recorded in Hodh El Gharbi (40%), followed by Adrar, Tagant, and Assaba (18–20%). Moderate rates were observed in Brakna and Trarza (12%), while lower rates were found in Gorgol, Guidimakha, Hodh El Charghi, and Dakhlet Nouadhibou (8–9%). Traces were detected in Inchiri (3%) and Tiris Zemmour (2%). These results suggest more intense viral activity in the southern regions, particularly in Hodh El Gharbi and Assaba, with signs of spread towards the center (Tagant) and the north (Adrar). The high seroprevalence observed in the main livestock farming areas underscores the persistent threat that RVF poses to food security and public health.

No statistically significant difference in seroprevalence was observed between sheep and goats, which is consistent with the results of Poueme et al. [30]. However, other studies have reported species-specific differences, such as higher seroprevalence in goats in Senegal [31] and higher rates in sheep in Egypt [32].

Older animals (over one year old) showed higher seropositivity (14.4%) than younger animals (7 to 12 months old) (11%), which is consistent with cumulative exposure profiles reported in other studies [33]. Nevertheless, the detection of antibodies in young animals in the southern, central, and northern regions suggests recent viral circulation. The predominance of females in the sample, likely due to a longer productive lifespan, complicates the interpretation of the results by sex, although their longevity may partly explain the higher seropositivity. Abortions were relatively rare (2%, or 14 adult females out of 688), but 5 of the 15 cases were seropositive, indicating a potential association with RVFV infection and warranting further investigation.

The cross-sectional nature of the study limits conclusions regarding temporal dynamics and recent transmission trends. The lack of entomological and environmental data restricts the analysis of ecological risk factors. Serological tests without longitudinal follow-up may underestimate recent infections, and uneven regional sampling may introduce spatial bias. Future studies should identify competent vector species and host preferences under various climatic conditions, conduct age-stratified serological surveys to clarify transmission dynamics, particularly in high-prevalence areas such as Hodh El Gharbi, establish longitudinal epidemiological surveillance systems integrating environmental, entomological, and serological data, and identify potential epicenter regions for better epidemic preparedness.

## 5. Conclusions

This study demonstrates that RVFV is widely circulating among small ruminants in Mauritania, with marked geographical heterogeneity and persistent foci such as Hodh El Gharbi. Although the overall seroprevalence is moderate (14.1%), the detection of antibodies in almost all of the studied regions confirms the endemic nature of RVF and the ongoing risk of re-emergence. The presence of antibodies in young animals suggests recent viral activity. Given the significant influence of environmental variability, livestock mobility, and livestock–human interactions, strengthening longitudinal serological monitoring, combined with entomological and ecological surveillance within the framework of the “One Health” approach, is essential to mitigate future epidemics and protect public health, animal health, and food security in Mauritania.

## Figures and Tables

**Table 1:** Characteristics of the studied small ruminants population.

Province of Origin	No. of Sampled Herds	No. of Goat (%)	No. of Sheep (%)	Total (%)
Hodh El Charghi	5	21	60	81 (9.3)
Hodh El Gharbi	6	22	57	79 (9.3)
Assaba	5	55	11	66 (7.8)
Gorgol	2	31	33	64 (7.5)
Brakna	6	33	47	80 (9.4)
Trarza	6	70	11	81 (9.5)
Adrar	6	34	52	86 (10.1)
Dakhlet Nouadhibou	4	9	42	51 (6.0)
Tagant	4	34	32	66 (7.8)
Guidimakha	6	9	70	79 (9.3)
Inchiri	5	62	4	66 (7.8)
Tiris Zemmour	4	48	2	50 (5.9)
Total (%)	59	428 (50.4)	421 (49.6)	849 (100.0)

**Table 2:** Seroprevalence of IgG antibodies against Rift Valley fever virus in the serum of small ruminants in Mauritania.

Parameters	No. of Tested Animals	Positive (%)	*cOR (95%CI)	*p*-Value
Species				
Goat	428	58 (13.6)	1	
Sheep	421	62 (14.7)	1.16 (0.74–1.82)	0.51
Sex				
Male	114	10 (8.8)	1	
Female	735	110 (15.0)	1.47 (0.70–3.07)	0.31
Age group (months)				
7–12	82	9 (11.0)	1	
>12	767	111 (14.5)	1.03 (0.49–2.19)	0.93
Province				
Hodh El Charghi	81	7 (8.6)	1	
Hodh El Gharbi	79	32 (40.5)	7.20	<10^−4^
Assaba	66	12 (18.2)	2.35	0.09
Gorgol	64	6 (9.4)	1.09	0.88
Brakna	80	9 (11.3)	1.34	0.58
Trarza	81	10 (12.3)	1.49	0.44
Adrar	86	17 (19.8)	2.60	0.05
Dakhlet Nouadhibou	51	4 (7.8)	0.90	0.87
Tagant	66	13 (19.7)	2.59	0.06
Guidimakha	79	7 (8.9)	1.04	0.94
Inchiri	66	2 (3.0)	0.33	0.17
Tiris Zemmour	50	1 (2.0)	0.22	0.16
Collection period				
Dry season	438	52 (11.9)	1	
Rainy season	411	68 (16.5)	1.38 (0.92–2.06)	0.12

*cOR: crude Odds ratio; 95% CI: 95% confidence interval of crude Odds ratio.

**Table 3:** Number of animals that aborted as a function of seropositivity.

Province of Origin	No. of Abortion	No. of Positive Animal	No. of Negative Animal
Hodh El Charghi	1	0	1
Hodh El Gharbi	7	4	3
Assaba	1	0	1
Gorgol	0	0	0
Brakna	0	0	0
Trarza	0	0	0
Adrar	4	0	4
Dakhlet Nouadhibou	0	0	0
Tagant	2	1	1
Guidimakha	0	0	0
Inchiri	0	0	0
Tiris Zemmour	0	0	0
Total (%)	15 (100)	5 (33)	10 (67)

## Data Availability

All data collected and analyzed in this study and the materials used are available from the corresponding author.

## Abbreviations

The following abbreviations are used in this manuscript:

ELISA	Enzyme-linked immunosorbent assay
FAO	Food and Agriculture Organization
ONARDEP	Office National de Recherches de Développement de l’Élevage et du Pastoralisme
RVF	Rift valley fever
RVFV	Rift valley fever virus
WHO	World Health Organization
WOAH	World Organization for Animal Health
